# Personalized Predictive Modeling of Subfoveal Choroidal Thickness Changes for Myopic Adolescents after Overnight Orthokeratology

**DOI:** 10.3390/jpm12081316

**Published:** 2022-08-15

**Authors:** Xiaohang Chen, Qiushi Li, Longqian Liu

**Affiliations:** 1 Department of Optometry and Visual Science, West China Hospital, Sichuan University, Chengdu 610041, China; 2 The Laboratory of Optometry and Vision Sciences, West China Hospital, Sichuan University, Chengdu 610041, China; 3 Department of Outpatient, West China Hospital, Sichuan University, Chengdu 610041, China; 4 Department of Ophthalmology, West China Hospital, Sichuan University, Chengdu 610041, China

**Keywords:** choroidal thickness, orthokeratology, myopia, multilevel model, adolescents, subfoveal choroidal thickness

## Abstract

The changes in subfoveal choroidal thickness after orthokeratology are crucial in myopia retardation; this study aimed to identify the risk factors that could be incorporated into a predictive model for subfoveal choroidal thickness (SFChT) that would provide further personalized and clinically specific information for myopia control. A one-year prospective study was conducted in the West China Hospital, Sichuan University. Basic information (age, gender, and height) was collected from all subjects. Initial spherical equivalent, axial length, intraocular pressure, central corneal thickness, and subfoveal choroidal thickness were measured, and the ocular environmental factors were also collected. All the measured parameters were recorded in the follow-up period for one year. After the analysis of univariate analysis, statistically significant factors were substituted into the multivariate three-level model. Thirty-three adolescents aged 8–14 years old were enrolled in this study; the results show that the subfoveal choroidal thickness in both eyes changed significantly after 12 months of lens wearing (*p*_R_ < 0.0001, *p*_L_ < 0.0001). The axial length was negatively correlated with the change in the SFChT after 12 months of lens wearing (r = −0.511, *p* = 0.002). After multilevel model analysis, the statistically significant factor was shown to have an important influence on the changes in the subfoveal choroidal thickness, which was the average near-work time. This suggested that the SFChT personal predictions can be made regarding changes in myopic adolescents after orthokeratology using the factor of daily average near-work time. Clinical practitioners will benefit from the results by obtaining a better understanding of the effects of orthokeratology on choroid and myopia progression.

## 1. Introduction

Myopia is one of the most prevalent disorders of the eye worldwide, and the prevalence of myopia in children has been on the rise [1]. This was particularly notable during the COVID-19 pandemic [2]. Among the numerous control methods for myopia [3], overnight orthokeratology lenses (Ortho-K lenses) have been widely used to remodel the corneal morphology and correct the refractive error in juveniles [3,4,5,6,7].

The choroid is located between the retina and sclera, which is a brown vascular layer with rich blood vessels [5,8]. A previous study reported an increase in choroid thickness (ChT) in the myopic defocus animal model [9]. Clinical studies found that the subfoveal choroidal thickness (SFChT) changed after orthokeratology [10,11]. Chen et al. [12] found increased ChT in subjects after three-week corneal shaping, which could be related to the effect of myopia control in orthokeratology lenses [13]. It is believed that the changes in the ChT and its physiological function are protective factors for myopia growth, affecting the toughness and ductility of the retina and sclera [14].

Therefore, it is important to find any factors that might affect the SFChT after orthokeratology and summarize the main factors to provide a reference for a clinical evaluation of the myopia-controlling effect in orthokeratology lenses.

As the data used in this study were independent, they were not suitable for traditional simple linear regression analysis. This method would be less accurate, and the results might be biased. Therefore, the multilevel model [15,16] and independent data analysis with hierarchical features were used to denote the changes into different levels, correct the parameter estimation error, and improve the confidence interval to facilitate more accurate and reliable conclusions.

## 2. Materials and Methods

### 2.1. Subjects

The study subjects were recruited at the contact lens clinic of West China Hospital from January 2017 to June 2018. The study was approved by the Ethics Committee on Biomedical Research, West China Hospital of Sichuan University (approval code: 2017-43). Before the onset of the study, informed consent was obtained from all subjects or guardians.

Patients with myopic children aged between 8 and 14 years were included in this study, and subjects had to wear primary orthokeratology lenses. Astigmatism after cycloplegic refraction was no higher than 1.50 D, and the initial spherical equivalent refraction (SE_0_) was less than or equal to 5.00 D. Patients with contact lens contraindications (such as dry eye, eyelid plate gland dysfunction, ptosis, and allergic rhinitis), surgical history of ocular trauma, allergies to cycloplegic drugs (compound tropicamide eye drops); strabismus, amblyopia, or other ocular diseases; and systemic diseases (such as diabetes, Down syndrome, and rheumatoid arthritis) were excluded from this study.

### 2.2. Ortho-K Lens Fitting

The subjects were instructed to insert and remove the lens; they also accepted the same training regarding lens care procedures after learning the lens fitting procedures (Table 1). To reduce the differences and effects between different brands and minimize the differences in various measurements due to different lens designs, all subjects in this study used VST design orthokeratology lenses (Euclid System Corporation, Sterling, VA, USA).

### 2.3. Measurements

Optical coherence tomography (ZEISS CIRRUS HD-OCT-5000) was used to measure the ChT of the subjects; the head position was fixed during the whole measurement. The “HD CROSS” test method and Enhanced Deep Imaging (EDI) mode were selected for standardized measurements [10,17].

**Figure 1:**
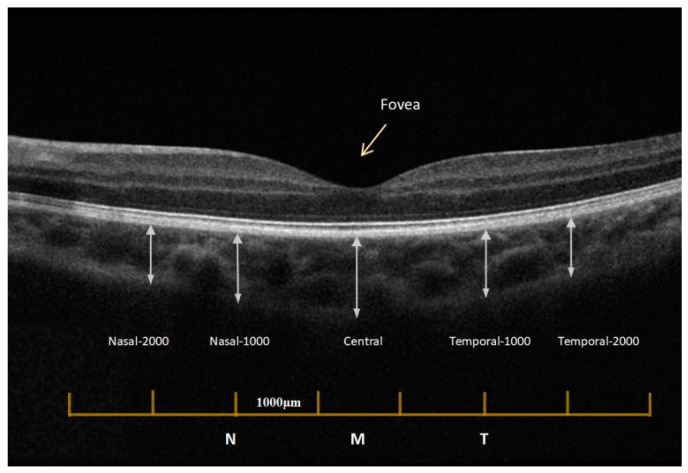
Choroidal imaging with EDI-OCT mode. EDI-OCT, Enhanced deep imaging optical coherence tomography. RPE, retinal pigment epithelium layer.

Similar to Yi et al. [18], both eyes were measured and analyzed by two independent practitioners of the study, zooming the images and using the instrument’s measuring tools to measure the choroidal thickness. Choroidal thickness was defined as the vertical distance from the RPE–choroid, choroidal–scleral boundaries, after removing the subject information and the test time (Figure 1). Two independent optometrists measured the thickness without any knowledge of the eye or other data, and the average of the two measurements was determined. The differences between the masked practitioners’ values were within 10% of the mean, as reported in Aydin et al., and all values remained within 8% in this study [19]. To exclude the effect of circadian rhythm on choroidal thickness measurements [20], all subjects had ChT measurements recorded between 9 am and 12 am, Beijing local time.

Other basic parameters, such as the axial length (AL), were measured using noncontact optical biometry (IOLMaster; Zeiss Meditec, Jena, Germany). The central corneal thickness (CCT) was measured using an endothelial microscope (SP-1P.; Topcon, Tokyo, Japan). Intraocular pressure (IOP) was measured using a noncontact tonometer (TX-20; Cannon, Tokyo, Japan). The initial spherical equivalent refraction (SE_0_) was measured by the phoropter (RT-600; Nidek Co., Ltd., Gamagori, Japan) after cycloplegia (Compound tropicamide; Xingqi Co., Ltd., Shenzhen, China). A resting period of at least 20 min was required before cycloplegia was achieved by administering the compound tropicamide four times into each eye at five-minute intervals.

### 2.4. Daily Ocular Environmental Factors

During the follow-up period, the environmental factors were collected through questionnaires and reported as follows (Table 2).

### 2.5. Statistical Analysis

The statistical software used in this study was SPSS 22.0, GraphPad Prism 8.3.0, MLwiN 3.00, and STATA 16.0. The test for normality was conducted using the Shapiro–Wilk test. Normally distributed data were expressed as the mean ± SD, while non-normally distributed data were expressed as the median (interquartile range). Qualitative data were expressed as the frequency (percentage).

Multiple comparisons were performed by repeated measures ANOVA (RM-ANOVA) with the Bonferroni correction for normally distributed data or Freidman’s test with Dunn’s correction for non-normally distributed data. *p* < 0.05 was considered statistically significant.

In this study, a multilevel model was used for comprehensive analysis. Using the SFChT as the primary outcome index, individual subjects, both eyes, and follow-up time level were the three levels used for the multivariate multilevel model analysis.

## 3. Results

### 3.1. Baseline Information

A total of 33 myopic children participated in this study. The basic data, measured before wearing the lens, are shown in Table 3.

**Figure 2:**
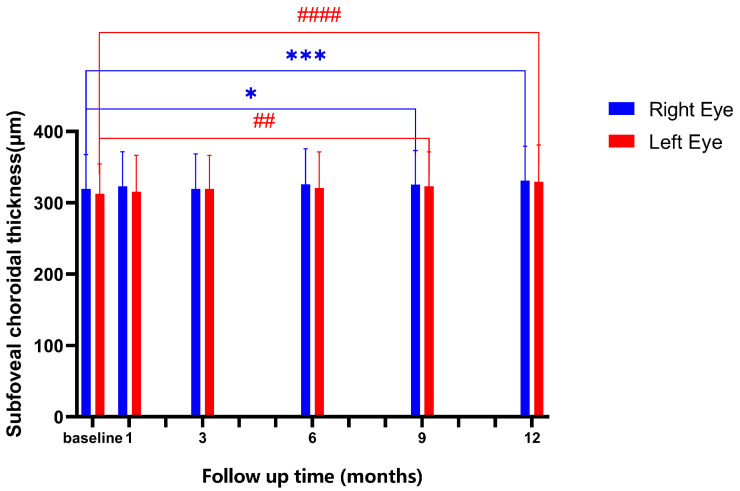
The binocular SFChT changes over the one-year follow-up. * represents *p* < 0.05; *** represents *p* < 0.001; all the data were from right eyes of the subjects. ## represents *p* < 0.01; #### represents *p* < 0.0001; all the data were from left eyes of the subjects. Bars represent mean; error bars stand for the standard deviation of the mean.

The choroidal thickness changes are shown in Figure 2. The baseline values of SFChT were 319.700 ± 48.010 μm in the right eye and 312.700 ± 41.940 μm in the left eye; there was no significance between the two eyes (*p* = 0.183). The results show significant changes in the SFChT in both eyes before and after lens wearing (*p*_R_ = 0.0001, *p*_L_ < 0.0001), and no difference between groups at 1 month, 3 months, and 6 months after lens wearing. Notably, at 9 months and 12 months after lens wearing, the SFChT changes were significant in both eyes (*p*_R-9_ = 0.024; *p*_R-12_ < 0.0001, *p*_L-9_ = 0.002; *p*_L-12_ < 0.0001).

**Figure 3:**
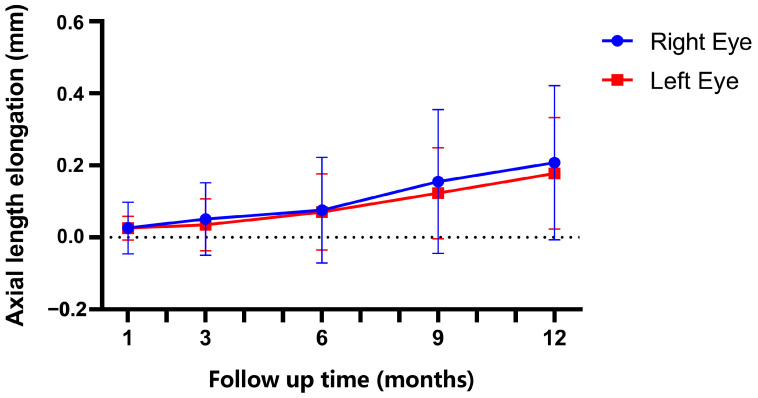
The changes in axial length in both eyes over the one-year orthokeratology lens wearing. Bars represent mean; error bars stand for the standard deviation of the mean.

The axial length values were 24.87 ± 0.79 mm in the right eye and 24.82 ± 0.82 mm in the left eye at the end of the follow-up. The data on the axial length elongation in both eyes are shown in Figure 3. The mean values of the axial length elongation after 12-month follow-up were: 0.208 mm in right eyes and 0.178 mm in left eyes; there was no significant difference in axial length elongation between the two eyes (*p* = 0.396). Significant changes were observed in AL in both eyes after orthokeratology (*p*_R_ < 0.0001 and *p*_L_ < 0.0001) and no difference between groups at 1 month, 3 months, and 6 months after lens wearing. However, AL changes were significant in both eyes at 9 months and 12 months after orthokeratology (*p*_R-9_ = 0.001; *p*_R-12_ < 0.0001, *p*_L-9_ < 0.0001, and *p*_L-12_ < 0.0001).

**Figure 4:**
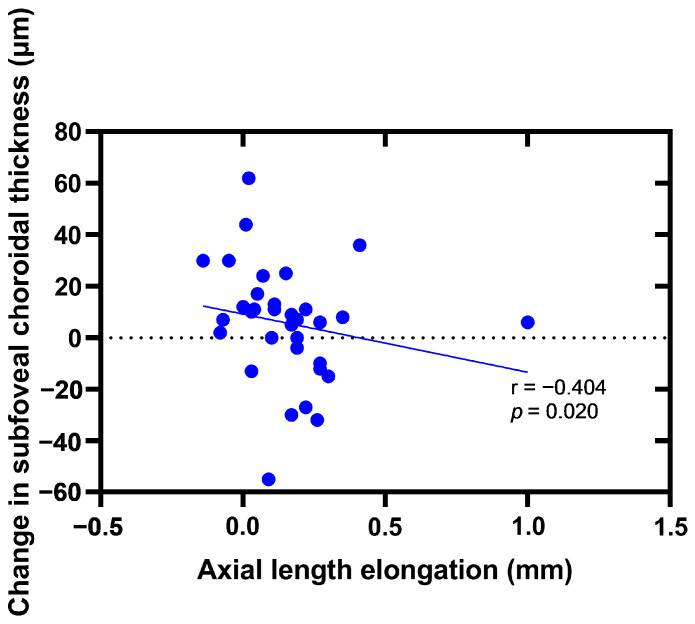
The correlations between the axial length elongation and the changes in the subfoveal thickness at nine-month follow-up.

**Figure 5:**
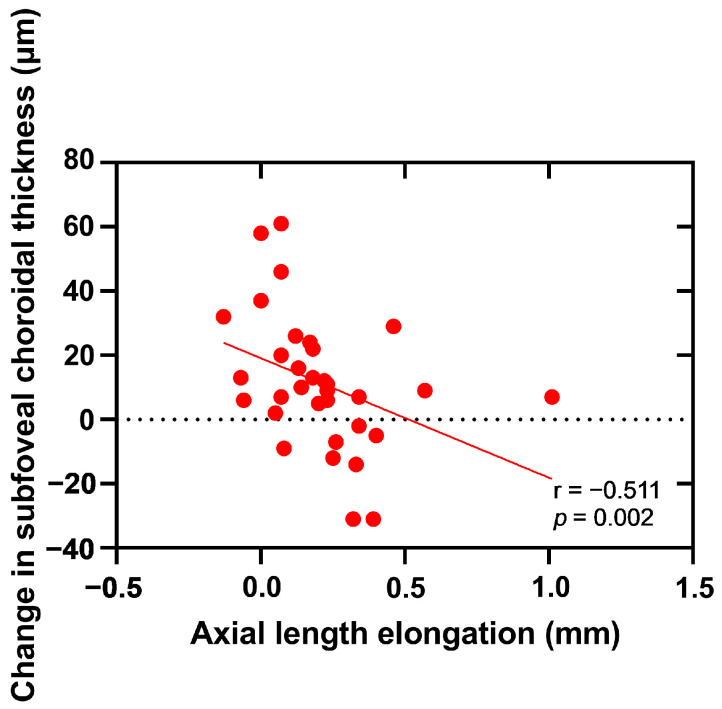
The correlations between the axial length elongation and changes in the subfoveal thickness at twelve-month follow-up.

Due to the correlation between the two eyes (r_AL_ = 0.966; *p*_AL_ < 0.0001, r_SFChT_ = 0.797; *p*_SFChT_ < 0.0001), we used the data of the right eye for the following correlation analysis. Figure 4 and Figure 5 report negative correlations between the axial length elongation and the changes in the subfoveal thickness at the 9-month follow-up and 12-month follow-up.

### 3.2. Establishing the Univariate Three-Level Model

First, the variables were calculated in the zero model for further analysis.

$${\mathrm{SFChT}}_{ijk}\sim \;N\;\left(XB,\Omega \right)$$

$${\mathrm{SFChT}}_{ijk}=\beta_{0ijk}cons$$

$$\beta_{0ijk}=322.169\left(7.759\right)+v_{0k}+u_{0jk}+e_{0ijk}$$

$$\left[v_{0k}\right]∼N\left(0,\;\Omega_{v}\right):\Omega_{v}=\left[1844.420\left(490.365\right)\right]$$

$$\left[u_{0jk}\right]∼N\left(0,\;\Omega_{u}\right):\Omega_{u}=\left[248.194\left(70.149\right)\right]$$

$$\left[e_{0ijk}\right]∼N\left(0,\;\Omega_{e}\right):\Omega_{e}=\left[219.110\left(17.058\right)\right]$$

*i*, *j,* and *k* refer to the follow-up period level, ocular level, and individual level, respectively. Ω refers to the variation in outcome indicators at the corresponding level. ~*N* (0, Ω) indicates that the variables in the box obey the following *N* distribution.

The risk factors were involved in the univariate three-level analysis shown in Table 4, and it is suggested that CCT and average near-work time were significant for SFChT after orthokeratology.

### 3.3. Establishing the Multivariate Three-Level Model

According to the analysis results, statistically significant factors were extracted into the multivariate multilevel model for further analysis (Table 4) using the following model:

$${\mathrm{SFChT}}_{ijk}=\beta_{0ijk}cons-212.330\left(115.576\right){\mathrm{CCT}}_{ijk}-3.425\left(1.725\right)T_{ijk}$$

$$\beta_{0ijk}=527.574\left(54.667\right)+v_{0k}+u_{0jk}+e_{0ijk}$$

$$\left[v_{0k}\right]∼N\left(0,\;\Omega_{v}\right):\Omega_{v}=\left[1922.879\left(510.917\right)\right]$$

$$\left[u_{0jk}\right]∼N\left(0,\;\Omega_{u}\right):\Omega_{u}=\left[252.745\left(70.673\right)\right]$$

$$\left[e_{0ijk}\right]∼N\left(0,\;\Omega_{e}\right):\Omega_{e}=\left[204.502\left(15.923\right)\right]$$

where *T* represents the average near-work hours per day for the subjects during the orthokeratology treatment.

It can be seen in Table 4 that a statistically significant factor with an important influence on the changes in the SFChT was the average near-work time. The coefficient of near-work time has a negative sign, where a higher value indicates a decrease in SFChT after orthokeratology.

## 4. Discussion

This study aimed to determine the individual and environmental factors that could affect choroidal thickness after orthokeratology over the one-year follow-up.

Over the one-year follow-up period, the study found that the SFChT became thicker in both eyes after orthokeratology, as was also reported in other studies [10,11,21]. Recent studies have suggested that the SFChT would change after orthokeratology lens wearing [22,23], with a significant thickening of the temporal choroid in the subfoveal area, but not in the nasal side [12]. However, Gardner et al. [24] found that the SFChT showed no significant change at 1 or 9 months of orthokeratology, probably because the sample size was small, with nine participants. In our study, it was found that the SFChT would not thicken continuously, a result consistent with the findings of the previous studies [11,25].

Previous studies have shown that, in normal physiological development, the choroid becomes thicker and the ocular axis extends [26,27]. When myopia is imminent, the choroidal thickness would become thinner [17,28] and is characterized by continuously thinning choroids in patients with progressive myopia. [29]. The reason for the SFChT changes after wearing an orthokeratology lens might be the choroidal blood vessels and choroidal blood flow [11,25]. A previous study showed that the thinning choroid could affect the choroid blood flow and cause scleral microenvironment hypoxia, which leads to an increase in axial length. Therefore, the SFChT would become thicker, and it could alleviate the growth in the scleral hypoxia environment to control the myopia progression [14].

In this study, the changes in the choroidal thickness after orthokeratology were negatively correlated with the axial elongation. In other words, the greater the change in SFChT, the smaller the axial elongation, and the better the effect of myopia control. This is similar to the study results of Li et al. [11]. However, in the study reported by Li et al., the researcher believed that the SFChT significantly changes after the first month of orthokeratology lens wearing, which was different from our study. In the present study, it is suggested that the SFChT does not change until the nine-month follow-up. One reason for this could be the sample selection; the subjects included in their study were adult wearers, whereas the subjects in our study were adolescent wearers, while changes in the ChT were also correlated with age [26,30].

The multilevel model was suitable for the variables in the study that are independent, with definite associations. Through the multilevel model analysis, it was suggested that, after wearing orthokeratology lenses, the SFChT was less affected by the initial refraction and age and more affected by the average near-work hours per day. It is reported that the strong accommodation response, which is thought to be related to myopia progression, was generally thought to be enabled by near work [31,32,33,34]. In addition, excessive near work is supposed to be related to a thinning choroid and decreasing choroidal blood perfusion [35,36]. Since the physiological function of the choroid is highly correlated with myopia progression [37,38,39], it can be speculated that the choroid could be an intermediate factor between near work and myopia progression [40,41,42].

However, some limitations in this study should be taken into consideration for further study. Patients were recruited from a geographically limited area, which is a limitation of this study. Another limitation was the relatively short follow-up period of one year, and a longer follow-up period is needed to validate this model and support our findings.

## 5. Conclusions

In this study, we measured the changes in the choroidal thickness before and after orthokeratology, and significant SFChT thickening was found after 9 months after orthokeratology, which is consistent with the changes in axial length elongation. We found that personalized predictions regarding subfoveal choroidal thickness changes in myopia adolescents after orthokeratology can be made using the factor of daily average near-work time. These findings represent further evidence that model-based personalized methods can be used to predict clinical ophthalmic outcomes, and clinical practitioners will benefit from the results by obtaining a better understanding of the effects of orthokeratology on choroid and myopia progression.

## Figures and Tables

**Table 1:** Orthokeratology lens fitting and aftercare procedures.

	Details
Before orthokeratology lens fitting	Learn the benefits and risks of wearing orthokeratology lenses
	Ensure the wearer and/or guardian provide full informed consent
	The proper lens application and removal instructions, including lens maintenance and care
	Evaluation of ocular health (including eyelid condition and the corneal endothelium) by slit-lamp biomicroscopy
	Good adherence to routine follow-ups
Orthokeratology lensfitting	Evaluate and record data on the following measurements: Refraction Ocular health Corneal topography
	Lens parameter selection and lens fitting evaluation
	Fluorescein evaluation
	Final parameter selection and lens customization
Aftercare procedures	Avoid storing the lenses and other accessories in the bathroom or other humid places
	Carefully check the lenses and identify if there are any noncompliant behaviors
	Evaluation of the cornea condition
	Measure other data mentioned in this study

**Table 2:** The relative ocular environmental factors and assignments.

Ocular Environmental Factors	Classification	Assignment
Average working distance per day (Distance)	<33 cm	0
	33–40 cm	1
	>40 cm	2
Average near-work hours per day (Time)	>5 h	0
	2–5 h	1
	<2 h	2
Average sleeping hours per night (Sleep)	≤7 h	0
	8 h	1
	>8 h	2
Average outdoor time (Outdoor)	<30 min	0
	30–60 min	1
	61–120 min	2
	>120 min	3

**Table 3:** Baseline data of the subjects.

Characteristics	Right Eye	Left Eye
Age (yr)	10 (9, 11)	
Sex (Male)	17 (51.5%)	
Height (cm)	142.200 ± 7.400	
SE_0_ (D)	−2.500 (−3.375, −2)	−2.500 (−3.250, −1.500)
IOP (mmHg)	15.700 (14, 16.800)	15.600 (13.700, 17.600)
AL (mm)	24.659 ± 0.827	24.640 ± 0.834
Average working distance per day (distance)		
<33 cm	1 (3.0%)	
33 cm–40 cm	32 (97.0%)	
>40 cm	0 (0%)	
Average near-work hours per day (time)		
>5 h	5 (15.2%)	
2–5 h	21 (63.6%)	
<2 h	7 (21.2%)	
Average sleeping hours per night (sleep)		
≤7 h	0 (0%)	
8 h	29 (87.9%)	
>8 h	4 (12.1%)	
Average outdoor time (outdoor)		
<30 min	1 (3.0%)	
30–60 min	17 (51.5%)	
61–120 min	10 (30.3%)	
>120 min	5 (15.2%)	

SE_0_, the initial spherical equivalent refraction; IOP, intraocular pressure; AL, axial length. Normally distributed data were expressed as mean ± SD, while non-normally distributed data were expressed as median (interquartile range). Qualitative data were expressed by frequency (percentage).

**Table 4:** Personalized predictive modeling of subfoveal choroidal thickness for myopia adolescents after orthokeratology.

Variables	Model				Variance at the Individual Level	Variance at Ocular Level	Variance at Follow-Up Period Level	−2 Log Likelihood Function
	Intercepts	Parameters						
		Value Effect	Wald	*p*				
Zero model	322.169	-	41.52	<0.001	1844.42 (490.365)	248.194 (70.149)	219.110 (17.058)	3480.598
Univariate analysis model								
Age	395.925	−7.582 (4.921)	−1.54	0.123	1711.064 (457.625)	248.194 (70.149)	219.110 (17.058)	3478.305
Sex	286.612	23.946 (14.955)	1.60	0.109	1701.194 (455.202)	248.194 (70.149)	219.110 (17.058)	3478.129
Height	566.410	−1.717 (1.021)	−1.68	0.093	1687.706 (451.891)	248.194 (70.149)	219.110 (17.058)	3477.886
SE_0_	311.378	−4.162 (5.545)	−0.75	0.453	1807.078 (481.954)	248.746 (70.372)	219.110 (17.058)	3480.040
IOP	336.177	−0.958 (0.497)	−1.93	0.054	1829.601 (487.449)	253.825 (71.484)	216.421 (16.849)	3476.915
CCT	501.771	−351.468 (102.292)	−3.44	0.001 **	1932.393 (513.555)	249.495 (70.122)	210.687 (16.408)	3469.079
Average working distance per day	325.391	−3.43 (3.01)	−1.14	0.255	1838.487 (488.933)	248.326 (70.149)	218.316 (16.996)	3479.302
Average near-work hours per day	326.332	−4.504 (1.736)	−2.59	0.009 **	1897.213 (503.809)	249.014 (70.147)	214.189 (16.676)	3473.967
Average sleeping hours per night	328.111	−5.711 (4.135)	−1.38	0.167	1862.436 (494.971)	248.436 (70.148)	217.656 (16.945)	3478.699
Average outdoor time	324.915	−1.855 (1.546)	−1.20	0.230	1862.231 (494.999)	248.385 (70.148)	217.965 (16.969)	3479.163
Multivariate multilevel analysis model	527.574		9.65	<0.001	1922.879 (510.917)	252.745 (70.673)	204.502 (15.923)	3459.3688
CCT		−212.33 (115.576)	−1.84	0.066				
Average near-work hours per day		−3.425 (1.725)	−1.99	0.047 *				

* represents *p* < 0.05; ** represents *p* < 0.01; SE_0_, the initial spherical equivalent refraction; IOP, intraocular pressure; CCT, central corneal thickness.

## Data Availability

The raw data supporting the conclusions of this article will be made available by the authors, without undue reservation.
